# Spatial iTME analysis of KRAS mutant NSCLC and immunotherapy outcome

**DOI:** 10.1038/s41698-024-00626-6

**Published:** 2024-06-19

**Authors:** Dan Zhao, Haiqing Li, Isa Mambetsariev, Tamara Mirzapoiazova, Chen Chen, Jeremy Fricke, Deric Wheeler, Leonidas Arvanitis, Raju Pillai, Michelle Afkhami, Bihong T. Chen, Martin Sattler, Loretta Erhunmwunsee, Erminia Massarelli, Prakash Kulkarni, Arya Amini, Brian Armstrong, Ravi Salgia

**Affiliations:** 1 Department of Medical Oncology and Therapeutics Research, City of Hope, Duarte, CA USA; 2 Integrative Genomic Core, Beckman Research Institute of City of Hope, Duarte, CA USA; 3 Department of Computational & Quantitative Medicine, Beckman Research Institute, City of Hope, Duarte, CA USA; 4 Department of Applied AI & Data Science, City of Hope, Duarte, CA USA; 5 Department of Human Oncology, University of Wisconsin, Madison, WI USA; 6 Department of Pathology, City of Hope, Duarte, CA USA; 7 Department of Diagnostic Radiology, City of Hope, Duarte, CA USA; 8 Department of Medical Oncology, Dana-Farber Cancer Institute, Boston, MA USA; 9 Department of Medicine, Harvard Medical School, Boston, MA USA; 10 Department of Surgery, City of Hope, Duarte, CA USA; 11 Department of Radiation Oncology, City of Hope, Duarte, CA USA; 12 Light Microscopy/Digital Imaging Core, City of Hope, Duarte, CA USA; 13 Present Address: Department of Gastrointestinal Medical Oncology, The University of Texas MD Anderson Cancer Center, Houston, TX USA

**Keywords:** Non-small-cell lung cancer, Cancer genomics

## Abstract

We conducted spatial immune tumor microenvironment (iTME) profiling using formalin-fixed paraffin-embedded (FFPE) samples of 25 *KRAS*-mutated non-small cell lung cancer (NSCLC) patients treated with immune checkpoint inhibitors (ICIs), including 12 responders and 13 non-responders. An eleven-marker panel (CD3, CD4, CD8, FOXP3, CD68, arginase-1, CD33, HLA-DR, pan-keratin (PanCK), PD-1, and PD-L1) was used to study the tumor and immune cell compositions. Spatial features at single cell level with cellular neighborhoods and fractal analysis were determined. Spatial features and different subgroups of CD68^+^ cells and FOXP3^+^ cells being associated with response or resistance to ICIs were also identified. In particular, CD68^+^ cells, CD33^+^ and FOXP3^+^ cells were found to be associated with resistance. Interestingly, there was also significant association between non-nuclear expression of FOXP3 being resistant to ICIs. We identified CD68^dim^ cells in the lung cancer tissues being associated with improved responses, which should be insightful for future studies of tumor immunity.

## Introduction

Kirsten Rat Sarcoma virus (*KRAS*) gene is one of the most commonly mutated oncogenes in lung cancer^1,2^. Targeting KRAS has been challenging until the discovery of the allosteric mutant-specific inhibition by covalent binding to the mutant G12C residue beneath the switch-II region, which locks it in the inactive guanosine diphosphate (GDP) bound status^3^. Positive results from clinical trials of the *KRAS*^G12C^ inhibitors, including Sotorasib (AMG510) and Adagrasib (MRTX849), led to approval by the US FDA for previously treated *KRAS*^G12C^-mutated advanced non-small cell lung cancer (NSCLC)^4–7^. Efforts are underway to target other mutant forms of KRAS, such as the development of MRTX 1133, a *KRAS*^G12D^ inhibitor with promising results^8^. Pan KRAS inhibitor RMC-6236 which binds Cyclophilin A, a chaperone protein, and active GTP-bound RAS (RAS ON inhibitor) is in phase 1 trial of patients with G12 mutations (NCT05379985). However, there are challenges including short duration of response, primary and secondary resistance to KRAS inhibitors. Both genomic and non-genomic mechanisms have been associated with resistance to KRAS inhibitors^9^. Interestingly, *KRAS*^G12C^ inhibitors can cause a pro-inflammatory tumor microenvironment (TME) with increased T cells, macrophages and dendritic cell infiltration^10^. *KRAS* is known to have immune modulatory effects and tumor microenvironment changes are critical for durable treatment responses^11,12^. Increased macrophage infiltration in the TME of MRTX1133-treated tumors in mice has been reported, and it has been shown that T cell immunity is essential for durable responses to KRAS inhibition^8^. KRAS inhibitor combination with immune checkpoint inhibitors (ICIs) is under clinical development^13^. Understanding the crosstalk between immunotherapy and KRAS-targeted therapies has great scientific and clinical significance.

ICIs are currently used as monotherapy or combination therapy in frontline and subsequent lines of therapy for metastatic NSCLC as well as in the neoadjuvant and adjuvant settings^14–19^. Primary and acquired resistance to ICIs are common with response rates of ~20% for monotherapy and of ~40% for combination therapy, while most patients eventually have progression of the disease^20,21^. Reports on the outcome of ICI treatment in *KRAS* mutated NSCLC are inconclusive due to heterogeneity and complexity^22–27^. The underlying mechanisms of resistance to ICIs are not fully understood. Co-mutations with *KRAS* have been thought to be primary molecular drivers that define immunological response, such as *KEAP1/STK11* mutations that are associated with reduced immune cells infiltration and resistance to immune checkpoint inhibitors^28,29^. With more KRAS inhibitors in clinical development and combination therapies of KRAS inhibition and immunotherapy, there is an unmet need to characterize the immunological features of patients with *KRAS* mutated lung cancer to facilitate translational studies.

It is essential to understand the immune tumor microenvironment (iTME)^30^. Recent development of single cell analytics with formalin-fixed paraffin-embedded (FFPE) tissue samples has provides a useful tool for dissecting the immunological features of TME^31–33^. In this project, we studied the lung tumor tissue of patients with NSCLC who had response or resistance to ICIs using image mass cytometry. Tumor cells and immune cells were studied at single cell level and spatial analysis of different cell types and cell neighborhoods (CNs). We hypothesized that the iTME was associated with treatment outcomes in patients with *KRAS* mutated NSCLC and we aimed to identify the specific iTME profile that determined response status.

## Results

### KRAS mutation and co-mutations with clinical outcome

**Fig. 1:**
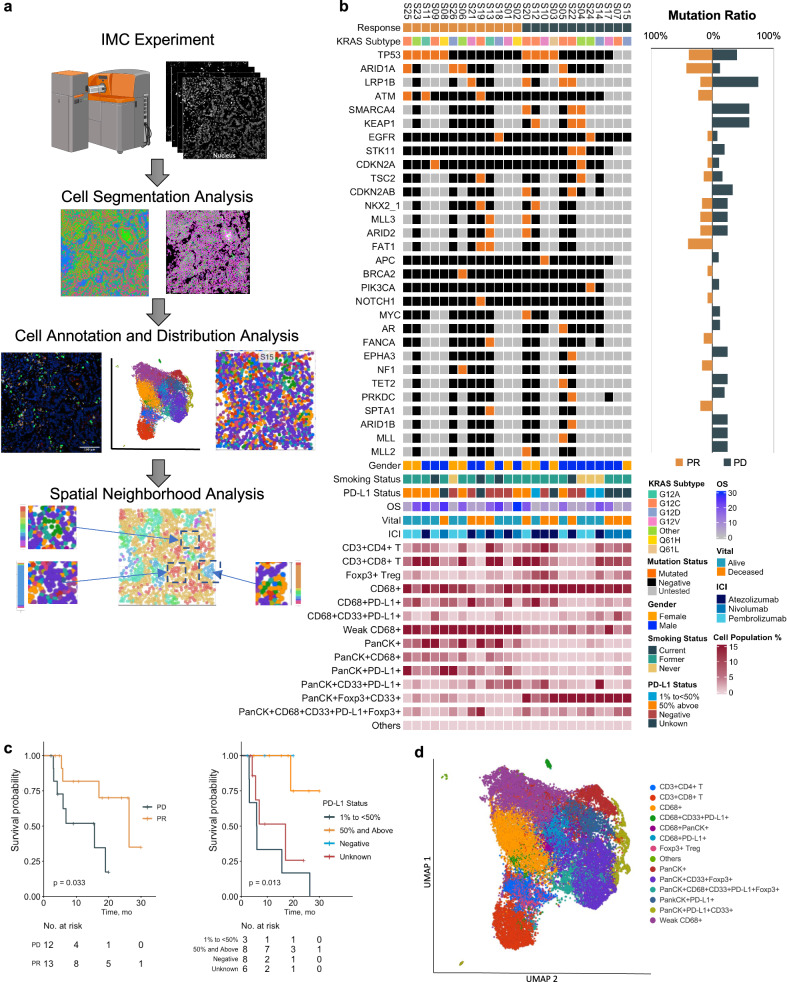
Overview of the patient characteristics and immune tumor microenvironment. **a** Workflow of the multiplex IMC imaging analysis of tumor tissues from the 25 patients with non-small cell lung cancer (NSCLC). Created with BioRender.com. **b** *KRAS* mutation, co-mutations, clinical features and main cell types of each patient grouped by response to immune checkpoint inhibitors (ICIs). **c** Overall survival (OS) by response to ICIs and Programmed Cell Death Ligand 1 (PD-L1) levels. **d** Uniform Manifold Approximation and Projection (UMAP) plot of the main cell types identified with color coded different groups of cells.

Figure 1a demonstrates the analysis pipeline for this study. Patients were stratified according to their treatment response status with 13 patients who responded to immunotherapy (responders) and 12 patients who did not respond to immunotherapy (non-responders) (Table 1). Clinical characteristics and landscape of *KRAS* mutations, comutations and immune features are summarized in Fig. 1b. The majority of the patients (21/25; 84%) received ICI monotherapy while a few patients (4/25; 16%) were treated with a chemotherapy-immunotherapy combination (Fig. 1 and Supplementary Table 1). There were no significant differences based on ICI treatment between responders and non-responders (Supplementary Table 2). Within the responder group, the *KRAS* mutations were G12C (*n* = 4), G12V *n* = 3), G12A (*n* = 2), G12D (*n* = 2), and Q61H (*n* = 2). Conversely, the non-responder group had G12C (*n* = 5), G12D (*n* = 3), G12V (*n* = 3), and Q61L (*n* = 1) *KRAS* mutations. The most common co-mutation was *TP53* (*n* = 9), followed by *ARID1A* (*n* = 4), *LRP1B* (*n* = 4), *ATM* (*n* = 3), and *SMARCA4* (*n* = 3). Notably, none of the responders had *KEAP1*, *STK11*, *CDKN2A/B*, nor *SMARCA4* mutation. All patients who had mutations in *KEAP1* (*n* = 3), *STK11* (*n* = 2), *CDKN2A/B* (*n* = 2), or *SMARCA4* (*n* = 3) were non-responders (Supplementary Table 3). All patients who had *ATM* (*n* = 3) or *FAT1* (*n* = 2) mutation were responders. The median overall survival (OS) of responders was 26.3 months versus 15.6 months for non-responders (*p* < 0.05). Programmed Cell Death Ligand 1 (PD-L1) levels were also associated with differences in OS in *KRAS* mutated patients. Patients who had PD-L1 levels at or above 50% had a significantly increased median OS (median OS not reached) than patients who were PD-L1 negative (median OS 17.0 months) and PD-L1 level at 1-50% (median OS not reached, 19.0 months using 95% low confidence interval, *p* < 0.05) (Fig. 1c). Compared to patients with PD-L1 at 1% and above, the hazard ratio (HR) of patients with negative PD-L1 was 9.55 (95% CI 1.05 ~ 87, *p* < 0.05). Within the 11 patients who had PD-L1 negative or 1–50%, 6 had progression of disease (54.5%) and 5 had partial response (45.5%). Within the 8 patients who had PD-L1 ≥ 50%, 6 patients (75%) were responders and 2 patients (25%) were non-responders (Fig. 1b).

**Table 1:** Patient characteristics

		PD	PR	*p*-value^a^
Age	Median (IQR)	67 (57-72)	65 (56-74)	0.757
Gender	Female	4 (33.3%)	6 (46.2%)	0.806
	Male	8 (66.7%)	7 (53.8%)	
Smoking Status	Never	3 (25.0%)	1 (7.7%)	0.364
	Current	1 (8.3%)	3 (23.1%)	
	Former	8 (66.7%)	9 (69.2%)	
Histology	Adenocarcinoma	10 (83.3%)	13 (100.0%)	0.308
	Squamous	1 (8.3%)	0 (0.0%)	
	Others	1 (8.3%)	0 (0.0%)	
Stage	IIB	0 (0.0%)	1 (7.7%)	0.511
	III	1 (8.3%)	2 (15.4%)	
	IV	11 (91.7%)	10 (76.9%)	
PD-L1	Negative	3 (25.0%)	5 (38.5%)	0.105
	1% to <50%	3 (25.0%)	0(0%)	
	50% and above	2 (16.7%)	6 (46.2%)	
	Unknown	4(33.3%)	2(15.4%)	

*IQR* Interquartile range, *PD* progression of disease, *PR* partial response.

^a^Chi-square test.

### Immune tumor microenvironment

To define the iTME, we profiled tumor samples from 25 patients using mass cytometry by time of flight imaging mass cytometry (IMC). The multiplex IMC data were converted to Tag Image File Format (TIFF image) for downstream cellular image analysis. Each cell was segmented using multiple markers to define the cell area and the background. Cells were clustered using Uniform Manifold Approximation and Projection (UMAP) with all markers and were annotated based on the cluster’s predominant marker profile. The cellular neighborhood was identified using the local cellular composition (Fig. 1a). The markers used to characterize the immune and tumor compartments of the iTME were: CD3, CD4, CD8a, FOXP3, PD-1, PD-L1, CD68, CD33, and pan-keratin (PanCK). From this analysis, 14 main cell types were identified, including CD4 T cells (CD3^+^ and CD4^+^), CD8 T cells (CD3^+^ and CD8^+^), CD68^+^ cells, CD68^+^CD33^+^PD-L1^+^, CD68^+^PancCK^+^, CD68^+^PD-L1^+^, FOXP3 T-reg cells (CD3^+^ FOXP3^+^), PanCK^+^, PanCK^+^CD33^+^FOXP3^+^, PanCK^+^CD68^+^CD33^+^PDL1^+^FOXP3^+^, PanCK^+^PDL1, PanCK^+^PDL1^+^CD33^+^ and CD68^dim^ (CD68 weakly positive, no other markers) cells. Cells without all the above specific markers were classified as “others” cell type (Fig. 1d). Single-cell measurements for all markers and cell spatial features were extracted from all images combined with the segmentation masks; single-cell level marker intensities of each sample were integrated using general linear model to remove the sample variation. The UMAP plot of cell colors was coded by the main cell types identified in the IMC experiment, including 14 main cell types based on the combination of the 11 biomarkers (Fig. 1d).

**Fig. 2:**
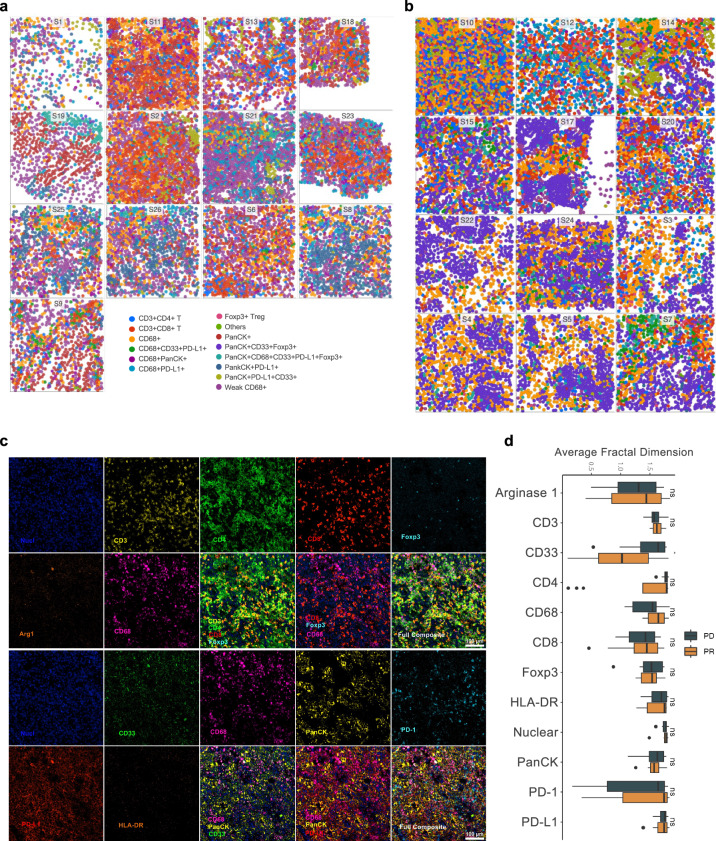
Cell spatial distribution, composition and fractal analysis in tumor tissues. **a** Cell type distribution color coded by the 14 different cell types across the tissue samples in responders. **b** Cell type distribution color coded by the 14 different cell types across the tissue samples in non-responders. **c** Representative tissue image of immune and tumor cell markers illustrated by IMC. The top two panels represent nuclei (blue) and markers of CD3 (yellow), CD4 (green), CD8 (red), FOXP3 (teal), Arg1 (orange), and CD68 (purple). The bottom two panels represent nuclei (blue) and markers of CD33 (green), CD68 (purple), PanCK (yellow), PD-1 (teal), PD-L1 (red), and HLA-DR (orange). CD68 was included in both panels because CD68 positivity was commonly seen in all samples. **d** Fractal analysis comparing individual cell marker distribution of average fractal dimension (FD) scores between responders and non-responders. *p < 0.05; ns not significant.

The individual cells were clustered based on marker expression using an unsupervised clustering algorithm, PhenoGraph^34^. The cell phenotype was annotated based on the expression intensity of all measured markers. We found different levels of intensity of CD68 markers in different cells with some cells being weakly positive for CD68 (CD68^dim^) while others were strongly positive (CD68^+^) (Supplementary Fig. 1 and Supplementary Fig. 2). For cells which were CD68 positive, the clustering algorithm identified CD68/CD33/PD-L1 triple positive, CD68/PanCK double positive, and CD68/PD-L1 double positive groups of cells. Interestingly, the CD68^dim^ cluster was distinctly detected in the responders rather than the non-responders (Fig. 2a, b). Five clusters of PanCK positive tumor cells were identified: PanCK positive, PanCK/PD-L1 double positive, PanCK/PD-L1/CD33 triple positive, PanCK/CD33/FOXP3 triple positive, and PanCK/CD68/CD33/PD-L1/FOXP3 positive (Fig. 2a, b). The phenotypic maps were colored to show the expression and spatial distribution of the individual markers and subtype cells (Fig. 2c). As illustrated in Fig. 2a (responders) and Fig. 2b (non-responders), there was enrichment of CD8^+^ T cells in responders, and enrichment of FOXP3^+^ Treg cells and PanCK/CD33/FOXP3 triple positive cells in non-responders. The cell marker spatial localization was analyzed using fractal analysis to quantify the spatial distribution of the individual cells using average fractal dimension (FD). We utilized FD to determine the morphologies of immune and tumor markers and quantify the difference between responders and non-responders. Large FD was commonly associated with a more complex spatial distribution and a plethora of short-term variations^35,36^. In our analysis, we found that immune cell markers including CD3, CD4, CD8, and CD68 were associated with slightly larger FD in responders as compared to non-responders. Interestingly, there was no difference in FOXP3, PD-1 or PD-L1 FD scores between responders and non-responders. However, CD33 showed a lower FD in responders as compared to non-responders (*p* < 0.05, Fig. 2d).

### Diverged immune cell phenotypes in responder versus non-responders

**Fig. 3:**
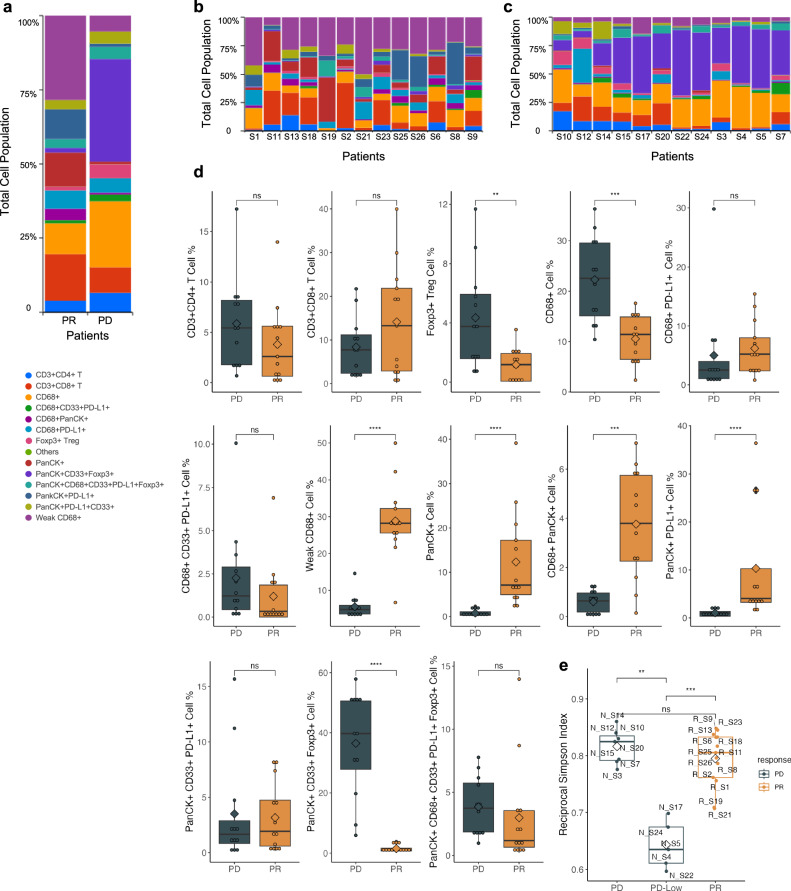
Distribution of main cell types between responders and non-responders. **a** Total composition of the 14 subtypes of cells in the responders and non-responders. **b** Cell subtype composition of each tissue sample of the 13 responders. **c** Cell subtype composition of each tissue sample of the 12 non-responders. **d** Box plots highlighting the differences in each cell subtype between responders and non-responders. **e** Diversity score of cell subtype population according to the mean value of diversity (Reciprocal Simpson index) between the responders and the non-responders. *<0.05., **<0.01, ***<0.001, ****<0.0001, ns not significant.

To evaluate the iTME diversity in the tissues, we measured the frequencies of each cell subtype composition between responders and non-responders across the 14 diversity markers, i.e., CD4 T cell, CD8 T Cell, CD68, CD68^+^CD33^+^PDL1^+^, CD68^+^PanCK^+^, CD68^+^PDL1^+^, FOXP3^+^Treg cell, others, PanCK, PanCK^+^CD33^+^FOXP3^+^, PanCK^+^CD68^+^CD33^+^PDL1^+^FOXP3^+^, PanCK^+^PDL1^+^, PanCK^+^PDL1^+^CD33^+^, and CD68^dim^ (CD68 weakly positive) (Fig. 3a). Within the responders, we found that CD68^dim^ cells (28.85%) were the major cell type followed by CD8^+^ T cells (14.14%) and PanCK^+^PDL1^+^ cells (10.32%). In the non-responders, PanCK^+^CD33^+^FOXP3^+^ cells (36.49%), CD68^+^ (22.31%), and CD8^+^ (8.39%) were the top three abundant cell types. CD68^dim^ cells were predominant (top cell population based on the percentage) in 12/13 responders. However, ≥5% CD68^+^ cells were detected in all tumors from the 12 non-responders (Fig. 3c). Also, 8/13 responders compared to only 4/12 non-responders had ≥5% CD8^+^ T cells in their tumor tissues. Interestingly, PanCK^+^CD33^+^FOXP3^+^ triple positive cells were detected in 12/12 non-responders with ≥5% abundance and it was predominant in 9/12 non-responders (Fig. 3b). 6/13 responders had ≥5% PanCK^+^PDL1^+^ cells (Fig. 3b). An increase in Foxp3^+^ T regulatory cells CD3^+^CD4^+^FOXP3^+^) was associated with non-responders (*p* < 0.01) (Fig. 3d). We found different subtypes of CD68 positive cells with CD68^dim^ cells associated with responders and enrichment of other CD68^+^ cells were correlated with non-responders (*p* < 0.001). Furthermore, PanCK^+^/CD33^+^/FOXP^+^ triple positive cells were enriched in non-responders (*p* < 0.001) and PanCK positive, CD68^+^/PanCK^+^ double positive, and PanCK^+^/PDL1^+^ double positive cells were associated with response to ICIs (*p* < 0.001; Fig. 3d). To measure the degree of iTME heterogeneity, diversity score of a tumor was calculated based on the 14 tumor and immune cell subtypes. We found that all responders had high diversity scores. The non-responders were stratified into two distinct subgroups, i.e., one subgroup with high diversity score and the other subgroup with low diversity scores (Fig. 3e).

### Differences in iTME landscape and cellular neighborhoods between responders and non-responders

**Fig. 4:**
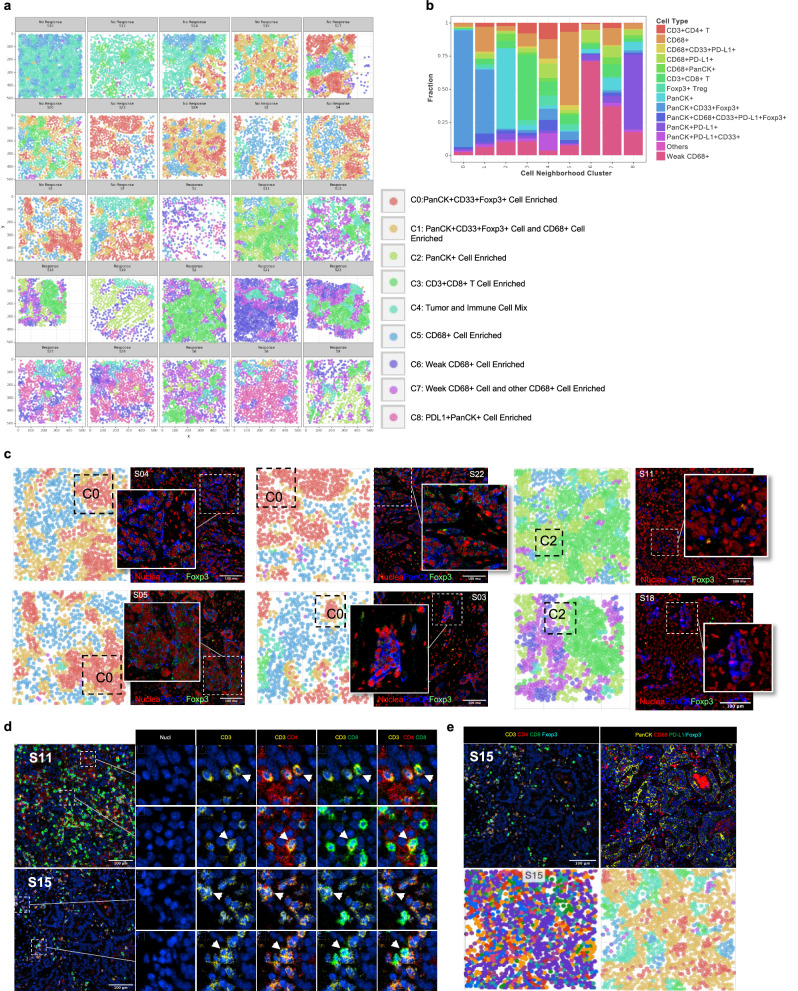
Cellular neighborhood characteristics and local neighborhood clusters. **a** The nine defined local cellular neighborhoods (CN) and the cell composition in each tumor sample (C0-C8). **b** Composition of the nine distinct cellular neighborhoods based on the frequencies of each cell types. **c** Color-coded cell neighborhood map as defined in the prior panel **a** corresponding to the IMC images from represented tissue samples S04 (non-responder), S05 (non-responder), S22(non-responder), S03(non-responder), S11 (responder) and S18(responder). **d** Magnified images of select tumor tissues of S11 (responder) and S15 (non-responder) showing the double positive CD4 and CD8 T cells. **e** High resolution image of seven cell type markers (top row), cell composition map (bottom left) and corresponding color-coded cell neighborhood map (bottom right) for S15 (non-responder).

To demonstrate and quantify the spatial heterogeneity of iTME, we visualized the tissues as aggregates of cellular neighborhood (CN), where a cellular neighborhood was defined as the local cellular composition around a cell. We identified 9 CNs (C0-C8). The CN clusters were named using the predominant cell types (Fig. 4a). The density of the individual CN was characterized by the proportions of each cell type that constituted most of the cells in the region (Fig. 4b). Figure 4a, b illustrate the CN composition and density of the iTME architecture within each tissue sample from the 25 patients. C0 was observed in majority of non-responders. It was enriched with PanCK^+^, CD33^+^ cells, and FOXP3^+^ T regulatory cells and lack of other cell types. Conversely, most responders were enriched for C3 and C6-7, which contained CD8^+^ T cells and CD68^dim^ cells. The analysis revealed distinct patterns in the iTME architecture where the individual CN shared little to no overlap and had distinct borders within the tissues in both responders and non-responders.

Following the abstract visualization analysis of CN C0, we found the marker FOXP3 being positive within the cytoplasm of the PanCK^+^ cell in four tumor tissues of non-responders (S03, S04, S05 and S22, Fig. 4c). We then reviewed C2 and found a distinct FOXP3^+^ signal which was localized in the nucleus in tissues of S11 (responder) and S18 (responder) (Fig. 4c). Upon further analysis of the localization of the markers and the cell nuclear signal, we identified CD4^+^CD8^+^ double positive T cell in two samples, one from a responder (S11) and one from a non-responder (S15) (Fig. 4d). To better visualize the spatial pattern of cellular neighborhoods and cell types, we demonstrated the detailed IMC images, cell type annotation in color images, and CN color-coding of tissue from a non-responder (S15) (Fig. 4d, e). Although, CD4^+^CD8^+^ double positive T cell was detected, they were uncommon and dispersed by the other cells such as PanCK^+^, FOXP3^+^, and CD68^+^ cells (Fig. 4e).

## Discussion

In this study, we conducted spatial iTME profiling using FFPE samples of 25 *KRAS* mutated NSCLC patients treated with ICIs. We studied the tumor and immune cell compositions and spatial features at the single cell level and by cellular neighborhoods. We found spatial features and the different subgroups of CD68 positive cells and FOXP3^+^ cells associated with response and resistance to checkpoint inhibitors.

In our study, 12 patients had a partial response (responders) and 13 had progression of disease (non-responders) to ICIs. Only 4 patients (16%) were never smokers and most patients were smokers, which was expected considering the association of *KRAS* mutation and tobacco smoking^37^. Consistent with other reports, *KEAP1*, *STK11*, *CDKN2A/B*, and *SMARCA4* mutations were associated with resistance to immunotherapy (Fig. 1)^28,29,38^. Our finding of ATM mutation being associated with responses was consistent with a large dataset report^39^. Others reported that *STK11* and *KEAP1* mutational inactivation resulted in fewer immune cells in *KRAS* mutated lung cancer^40^. We did not have enough samples to compare *STK11/KEAP1* mutated versus non-mutated patients for the iTME analysis. *KRAS*-mutated cancers are heterogeneous with different mutation allele subtypes and co-mutations^41–43^. *KRAS*^*G12D*^ mutation has been reported to be more immunosuppressive. However, we found no statistically significant difference between different *KRAS* mutation alleles regarding responses to immunotherapy and iTME features, possibly due to our limited sample size (Supplementary Table 4)^44^. We found patients with PD-L1 level ≥50% had significantly longer survival than patients with PD-L1 < 50%, which suggests further larger studies and prospective clinical trials are required to determine the impact of PD-L1 expression in *KRAS*-mutated patients. Our data suggests that *KRAS*-mutated patients with PD-L1 ≥ 50% may benefit from the use of immunotherapy as compared to those with PD-L1 < 50%. Our results are consistent with the emerging data on preference of chemoimmunotherapy over immunotherapy alone for *KRAS* mutated patients who had PD-L1 < 50% or with co-mutation of *KEAP1/STK11*^45^.

We compared the responders and non-responders to identify the differences of the population distribution of cell subtypes in iTME. It is well known that CD8^+^ T cells are associated with response to ICIs^46^. Not surprisingly, there were increased CD8^+^ T cells in the responders in the present study (Figs. 2 and 3). Tumoral immune cell infiltration especially tumor-infiltrating lymphocytes (TILs) are important for immunotherapy but there were tumors which had abundant TILs but not responding to immunotherapy^47–49^. We found high diversity, more subtypes of immune cells, in responders but not in the non-responders (Fig. 3e). The ones with low diversity could be the immune desert type tumors, that have fewer types of immune cells detected^50^. This is consistent with the transcriptomic data of two distinct transcriptional states of tumor microenvironmental signature with resistance to ICIs^39^. Our data suggested an immune suppressive environment in non-responders despite the presence of immune cells. The lack of functional immunity against cancer has not been fully understood. The functional status of myeloid cells is critical for cancer immunity, responses and resistance to ICIs^51^. We found different types of CD68 positive cells based on the intensity of the CD68 (weakly positive and strongly positive cells). The CD68 weakly positive cluster (CD68^dim^) was detected in the responders but not in the non-responders (Fig. 2a, b). CD68 is commonly used as a monocyte/macrophage marker. However, CD68 is also expressed in non-myeloid cells such as lymphoid cells, fibroblasts and tumor cells^52^. There is a lack of consensus in the human macrophage markers panel for staining. It is well known that the functional status of macrophages is context-dependent^53^. We suspect M1 versus M2 macrophages definition could be oversimplified and the spectrum of macrophage status could be continuous rather than dichotomous. There could be many subsets of CD68 expressing cells with distinct functions. In our study we found a group of cells with lower levels of CD68 expression (CD68^dim^ cells) distinguished themselves from those with higher expression of CD68. The CD68^dim^ cells identified in the present study are not well-defined and warrant further analysis. We found CD68 positive cells (not CD68^dim^) and PanCK^+^/CD33^+^/FOXP3^+^ cells being associated with resistance to ICIs (Fig. 3d), which was generally in agreement with the complexity of the myeloid cells and the context dependent functional spectrum of myeloid derived suppressor cells (MDSCs)^54,55^. Tumor cells expressing immune markers could be part of the mechanism of immune escape in the cancer immune cycle. We found two different patterns of FOXP3 expression, i.e., nuclear expression which was consistent with T reg cells and cytoplasmic expression of FOXP3 (Fig. 4c). Cytoplasmic FOXP3^+^ tumor cells were reported previously, and they were associated with worse prognosis in patients with breast cancer^56^. Conversely, significantly elevated FOXP3^+^ T reg densities were found in responders compared to non-responders in melanoma by IMC analysis of TME which could mediate tumor rejection after the ICIs^57^. We found FOXP3^+^ expression being associated with resistance to ICIs (Fig. 3d). Our result was consistent with the findings of enriched CD68^+^ macrophages and FOXP3^+^ cells in ICI refractory NSCLC^58,59^. Targeting FOXP3 by the PRMT5 inhibitor could be promising in lung cancer treatment^60–62^.

Interestingly, we found CD4^+^CD8^+^ double positive T (DPT) cells in the *KRAS* mutated NSCLC tissues (Fig. 4d). The RAS/MAPK signaling pathway is critical for thymocytes differentiating from CD4-CD8-double negative to CD4^+^/CD8^+^ DPTs and for positive selection of DPTs into CD4^+^ or CD8^+^ single cells by T cell receptor(TCR)^63,64^. *KRAS* knockout is embryonically lethal and *KRAS*^*G12D*^ knockin mutation caused CD4^+^/CD8^+^ double positive T lymphoblasts leukemia in the mice model^65,66^. Little is known about CD4^+^/CD8^+^ DPT cells found in peripheral tissues (~5%) but they were associated with peripheral immune tolerance and outcome of cancer^67–73^. Interestingly, CD4^+^/CD8^+^ DPT cells were identified and were found to form dense compartments that were highly correlated with responders and effector T cells functional gene expression in melanoma^57^. It has been reported that effector/memory T cells and memory/early activated CD8^+^ T cells generated after ICIs were associated with TCR diversity and response to ICIs while the origin of these cells was not clear^74^. The CD4^+^CD8^+^DPT cells were proposed to be reprogrammed from CD8^+^ T cells since activated CD8^+^ could acquire CD4 expression and CD4^+^CD8^+^DPTs had effector/memory phenotype and self-renewal capacity^69,70,73,75–77^. Future studies on *KRAS* and peripheral DPT cells with a larger population might be insightful for lung cancer immunotherapy.

Immune architectures are associated with cancer outcome^78^. Heterogeneity of immune cell function may exist beyond the abundance and intensity of expression markers, and the spatial distribution of the cells is important. In addition to analysis at the single cell level, we also conducted spatial analysis of the cellular neighborhoods (CNs). We identified 9 CNs (C0-C8) (Fig. 4a) and the iTME architectures differed between responders and nonresponders (Fig. 4a, b). Similarly, a large cohort study evaluating immunotherapy response in triple-negative breast cancer using spatial IMC analysis showed a similar phenomenon of 16 TME cell phenotypes associated with response most notably CD8^+^TCF1^+^T cells being the strongest predictor of overall response^79^. The T cell stemness and priming by macrophages is critical for ICI treatment effects. Our panel focused on the interaction of myeloid cells and T cells within cancer. Our real-world data for patients treated with ICIs provides additional information for therapeutics development targeting myeloid cells and reducing T reg in lung cancer. In our study, C0 which was enriched with PanCK^+^, CD33^+^ cells, and FOXP3^+^ T regulatory cells was observed in majority of nonresponders (Fig. 4b). However, most responders were enriched with C3, C6-C7 which contained mostly CD8^+^ and CD68^+^ cells (Fig. 4a, b). A larger cohort study evaluating immunotherapy response in lung cancer patients using differential IMC markers, identified CN23 neighborhood, consisting of five markers CD14, CD16, CD94, αSMA and CD117, most significantly associated with overall survival suggesting specific neighborhood interactions may have prognostic value^80^. Interestingly, C0 usually located inside the C1 with C1 being around the borders of the C0 (Fig. 4a). C1 contained CD68^+^ cells in addition to the PanCK^+^, CD33^+^ cells, and FOXP3^+^ T regulatory cells (Fig. 4b, c). In our FD analysis, we found that immune cell markers including CD3, CD4, CD8, and CD68 were associated with slightly larger FD in the responders as compared to the non-responders and CD33 showed a lower FD in responders (*p* < 0.05, Fig. 2d). FD is useful for describing and quantifying the morphology and architecture of tumors^35^. Larger FD is associated with geometrical complexity and irregularity of shapes and patterns^36^. Consistent with previously reported results, patients with enriched CD4+ and CD8 + T cells interacting with tumor cells were associated with better outcome and the myeloid component/M2 macrophage were critical for T cells^80,81^. The enrichment of FOXP3 + T reg were associated with worse survival and spatial interaction of suppressive myeloid cells and T regs were prominent in more aggressive tumors^80^. These results indicate that the architectures of boundary interaction of compartmentalized tumor CN and macrophage CN may suppress the T cell immunity and facilitate the peripheral immune tolerance of tumor. It is unclear how the architectures of tumor cells and immune cells were regulated. Future studies in spatial cancer immunology are warranted.

There were limitations to this study. First, there were inherent limitations associated with its retrospective study design as cofounding variables such as patients’ age, sex, clinical staging, prior treatment regimen, etc. may not be controlled. Second, this was a single institution study with limited sample size and availability of tissues. We did not have paired tissue samples before and post ICIs treatment for comparison nor peripheral blood/lymph node samples for profiling of systemic/reginal immune cells. Nevertheless, this descriptive retrospective analysis had its merits as it provided a real world clinical and molecular information on *KRAS* mutated lung cancer patients treated with ICIs. We provided multiplex immune profiling of 11 markers simultaneously by the IMC platform using FFPE tumor tissue samples. We found CD68^+^ macrophages, MDSCs and FOXP3^+^ cells being associated with resistance to ICIs. We also identified a population of CD68 weakly positive (CD68^dim^) cells in the lung cancer tissues that being associated with improved responses, which may be insightful for future studies of tumor immunity.

## Methods

### Patients

Archived lung cancer tissue samples were obtained under IRB 17281 in accordance with City of Hope IRB and guidelines of Declaration of Helsinki. The City of Hope IRB granted a waiver of informed consent under 45 CFR § 46.116 based on determination that this research meets the following requirements: (i) the research involves no more than minimal risk to the subjects; (ii) the research could not practicably be carried out without the requested waiver; (iii) the waiver will not adversely affect the rights and welfare of the subjects. Initially we identified 87 *KRAS*-mutated NSCLC patients treated at City of Hope. Patients were selected through retrospective chart review and EMR schedule review on the basis of having a *KRAS*-mutation detected by NGS at City of Hope^38^. Following this study, the 25 patients with *KRAS* mutated NSCLC treated with ICIs at City of Hope had tissue samples available for this study and were selected based on tissue availability and response status of responders vs non-responders. Twenty-three patients had lung adenocarcinoma, 1 patient had lung squamous cancer and 1 patient had large cell carcinoma. The clinical tissue specimens were collected prior to initiation of treatment at time of diagnosis and were obtained retrospectively. Response status was evaluated clinically and radiologically. Fifteen (60%) were male and 10 (40%) were female. Four (16%) were current smokers, 17 (68%) were former smokers, and 4 (16%) were never smokers. Of the 25 patients, 21 patients had a single *KRAS* mutation and 4 patients were found to have multiple *KRAS* alterations. Eight (32%) patients’ *KRAS* mutations were G12C, 4 (16%) were G12V, 2 (8%) were G12A, 4(16%) were G12D, 2(8%) were Q61H, 1(4%) were Q61L, 4 were others (1 patient with G12V/G12D/G12R and 3 patients with both *KRAS* mutation and *KRAS* amplification). The tumor stage was categorized using the American Joint Committee on Cancer (AJCC) TNM criteria and 21 patients had stage IV lung cancer, with 3 patients with Stage III and 1 patient with Stage II. Among the 25 patients, 12 had partial responses (PR) and 13 had progression of disease (PD) based on the iRECIST criteria^82^.

### Tissue molecular profiling of tumor and immune cells

FFPE slides were stained with heavy metal-conjugated antibodies for imaging mass cytometry (IMC) using the Fluidigm Hyperion™ imaging system (Fluidigm Corporation, South San Francisco, CA, USA). An eleven-marker panel (CD3, CD4, CD8a, FOXP3, CD68, arginase-1, CD33, HLA-DR, Pan-Keratin (PanCK), PD-1, and PD-L1) of tumor stromal and immune markers was assessed and quantified cellular relationships in the tumor microenvironment. The slides were simultaneously stained by multiple antibodies and analyzed by IMC technology. Slides were dewaxed in xylene and hydrated in descending grades of ethanol (100%, 95%, 80%, 70%, 5 min each). The slides were then incubated in heated Tris/EDTA antigen retrieval solution (Dako PH9, Agilent, Santa Clara, CA) for 30 min and blocked with 3% BSA solution for 45 min at room temperature after washed with double distilled water (ddH2O) and Dulbecco’s Phosphate Buffered Saline (DPBS). A custom panel of 11 metal-label antibodies (Supplementary Table 5) was generated according to the protocol from Fluidigm. The slides were stained with the antibodies cocktail in 0.5% BSA overnight at 4 ˚C in hydration chamber. After being stained with antibodies, slides were washed with 0.2% Triton-X in DPBS and then stained with Ir-Intercalator (1:600, Fluidigm) in DPBS for 30 min at room temperature. The stained slides were sent for imaging analysis after rinse and air dry. Tiled images were taken from the prepared slides on a Zeiss Observer Z1 using a 5x/0.16 NA objective and stitched using Zeiss ZEN Blue software (Carl Zeiss Microimaging). The images were oriented using Image-Pro Premier 9.3.3 (Media Cybernetics, Baltimore, MD) to assist in locating and accurately selecting the appropriate regions of interest (ROIs) for laser ablation and data acquisition using the Hyperion Imaging Cytometer (Fluidigm) on ROIs of 500um x 500um. Representative intertumoral area (avoiding boundaries between stromal and tumoral areas) was selected for region of interest (ROI) using the corresponding H&E histology slides.

The data of each marker were exported as TIFF format for downstream quantification. A combination of markers including CD3, CD4, CD8, FOXP3, CD68, arginase-1, CD33, HLA-DR, Pan-Keratin (PanCK), PD-1, and PD-L1 was used to generate cell segmentation masks, which defined the region of each individual cell and the background area on each image. Cell segmentation was performed using CellProfiler based on the mix of the markers images^83^, leveraging a probability image generated for cell nuclei, cell membranes, and background through a machine learning approach implemented in ImageJ’s Trainable Weka Segmentation plug-in^84,85^. The model was trained using a combination (Fig. 1a and Supplementary Fig. 3A) of cell membrane marker images to identify the cell membrane region, cell nuclei marker images to delineate the cell nuclei region, and background regions for background identification. Subsequently, this trained model was applied to all samples to produce the probability image (Supplementary Fig. 3B). CellProfiler was then employed to segment individual cells from the probability image. Nuclei were identified as primary objects based on the nuclei probability using IndentifyPrimaryOjbect feature, and cytoplasm and cell membrane were delineated by expanding identified nuclei to the border between the cell cytoplasm/membrane and background using IdentifySecondaryObject feature with the propagation method^86^. The identified nuclear and cellular boundaries were exported as Cell segmentation masks in text image TIFF format files (Supplementary Fig. 3C) for signal quantification and neighborhood analysis.

Accurate cell counts and identification of spatial relationships including co-localization and cell clustering were analyzed using HistoCAT software and Partek® Flow® software^87,88^. The IMC data underwent pre-processing via HistoCAT software for arcsinh transformation. Data from all 25 patients were integrated using a general linear model to eliminate batch effects and the same signal threshold was used across the patient tissue. Mutated Genes are extracted from clinical reports as binary data. The Field of view of the tissue is 500 um × 500 um (except sample 18). We detected total of 27214 cells with an average of 1089 cells per patient. We classified those cells into 13 subtypes based on their marker intensity profile (the “other” cells were not included in the analysis due to a lack of markers). The amount of minimal abundant cells was 2.1% of total cells (Supplementary Table 6). We included as many as possible patient samples for each phenotype to increase the detection power. Meanwhile, a novel approach, dubbed Sensei, was utilized to determine whether the number of samples and the number of cells were sufficient to ascertain changes between two groups^89^. Single-cell measurements for all markers and cell spatial features were extracted from all images combined with the segmentation masks; single-cell level marker intensities of each sample were integrated using general linear model to remove the sample variation. Multidimensional reduction was performed via Uniform Manifold Approximation and Projection (UMAP)^90^, allowing for visualization of multiplexed measurement within two-dimensional planes. An unsupervised clustering algorithm, PhenoGraph was used to classify the cell phenotypes based on the abundances of all measured markers^34^. The cell’s spatial features including size, shape, and cell location were not included for the cell cluster analysis. The cell population (percentage) difference between PR and PD patients was tested using R’s stat package Wilcoxon test (version 3.6.2) and the results were visualized using ggplot2 package (version 2.3)^91,92^. Cell population diversity was analyzed with cell subtype percentages using R diverse package (Ver 0.1.15)^93^. The high and low diversity was determined using the mean of the simpson diversity index in each response groups^94^.

### Neighborhood spatial analysis and fractal analysis

Neighborhood Spatial Analysis was performed using a cell spatial analysis pipeline which determined the cellular composition within a 30-μm radius around individual cells and performed unsupervised k-means clustering (Python Scikit-learn, version 0.21.2) followed by manual annotation using the main cell population in each cluster^95^. Fractal Dimension (FD) of each marker was analyzed using the box count method implemented in FIJI Fraclac Plug^96^. Each biomarker’s IMC image was binarized first and the FD was analyzed. The results were summarized using the average FD for each cohort. To compare the FD between the two cohorts in this study, each Biomarker’s FD was normalized based on its tumor cell biomarker (PanCK).

### Statistical analysis

The clinical and molecular features including tissue immune cell signatures were analyzed and compared between the responders (*n* = 13) and non-responders (*n* = 12). The OS was defined from the start of ICIs until death due to any cause or last follow up. The association of clinical and molecular features with OS was analyzed by univariate COX proportional hazards model independently. PD-L1 expression was categorized as negative (<1%), 1%–<50% and ≥50%. The Kaplan–Meier method was used to estimate OS and the Log-rank test was used to compare the survival curves. The association of immune cell populations and expression level of immune markers with clinical outcomes (OS, responses) were determined by Wilcoxon tests, Chi-square tests and logistic regression. Statistical analyses and data visualization were performed using R (open source for statistical computing and data visualization). All tests were two-sided and *P* < 0.05 was considered statistically significant.

### Reporting summary

Further information on research design is available in the Nature Research Reporting Summary linked to this article.

### Supplementary information

Supplementary Information

Reporting Summary

## Data Availability

De-identified data is available from the corresponding author upon reasonable request.
