# Supplementary material for: Spatial iTME analysis of KRAS mutant NSCLC and immunotherapy outcome
Source: NPJ Precis Oncol. 2024 Jun 19;8:135. doi: 10.1038/s41698-024-00626-6 (PMC11187132; doi:10.1038/s41698-024-00626-6)
Supplement: Supplementary file 1 — Supplementary Information [file 41698_2024_626_MOESM1_ESM.pdf]

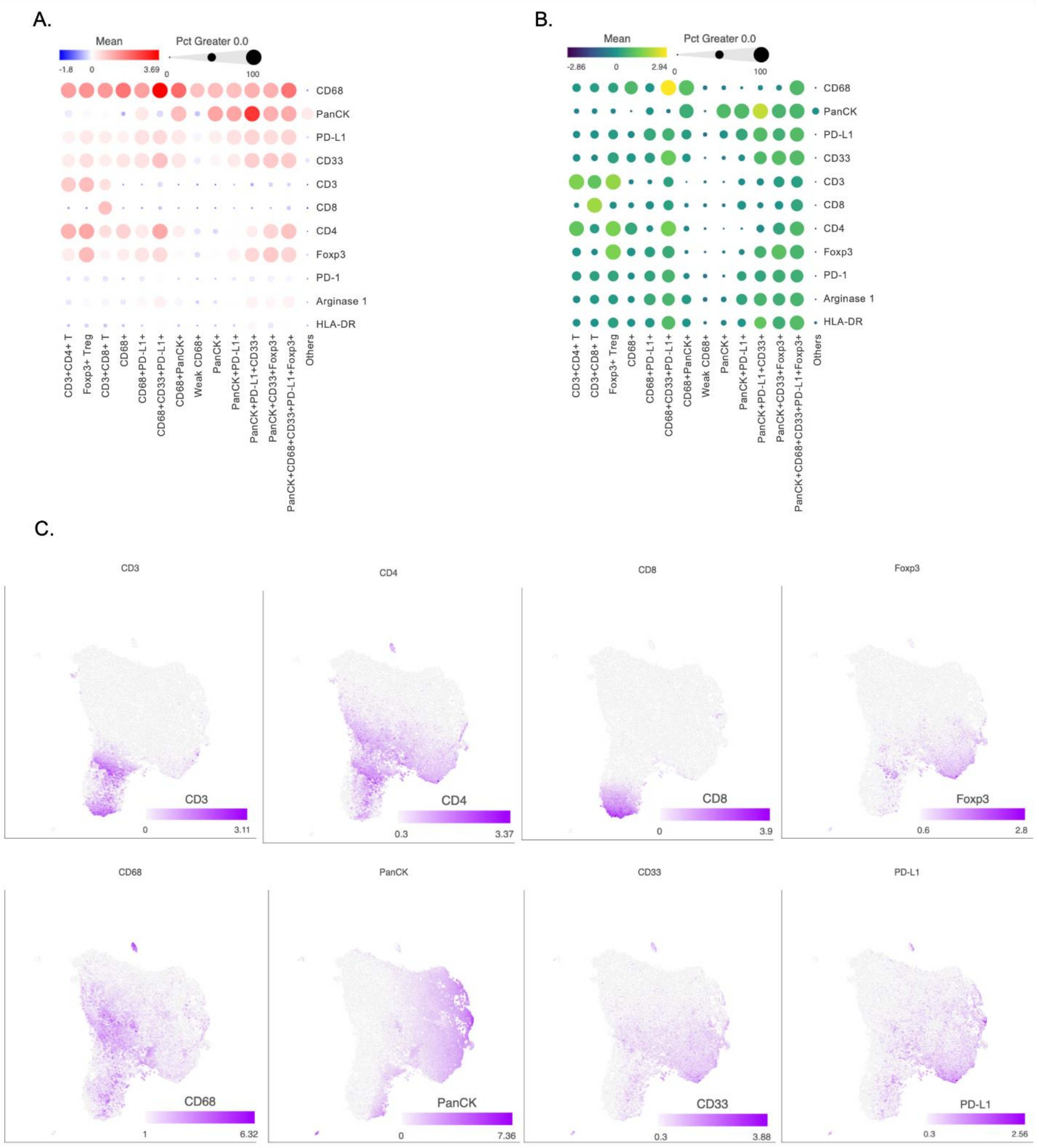

Supplemental Figure 1. Marker Intensity and Cell Annotation

A. Marker intensity per cell type. **B**. Z-score of marker intensity per cell type. **C**. Uniform Manifold Approximation and Projection (UMAP) color by marker intensity per cell.

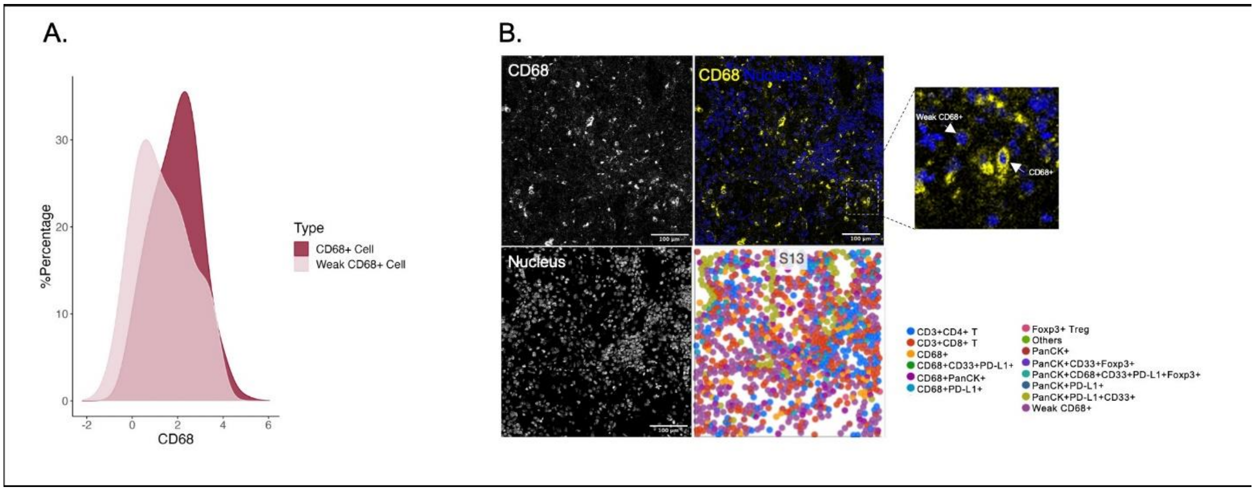

Supplemental Figure 2. The CD68 intensity for CD68+ cell and weak CD68+ cell

A) The CD68 Intensity for CD68+ cell and Weak CD68+ Cell; **B)** The CD68+ cell and Weak CD68+ cell on the IMC images and cell annotation map.

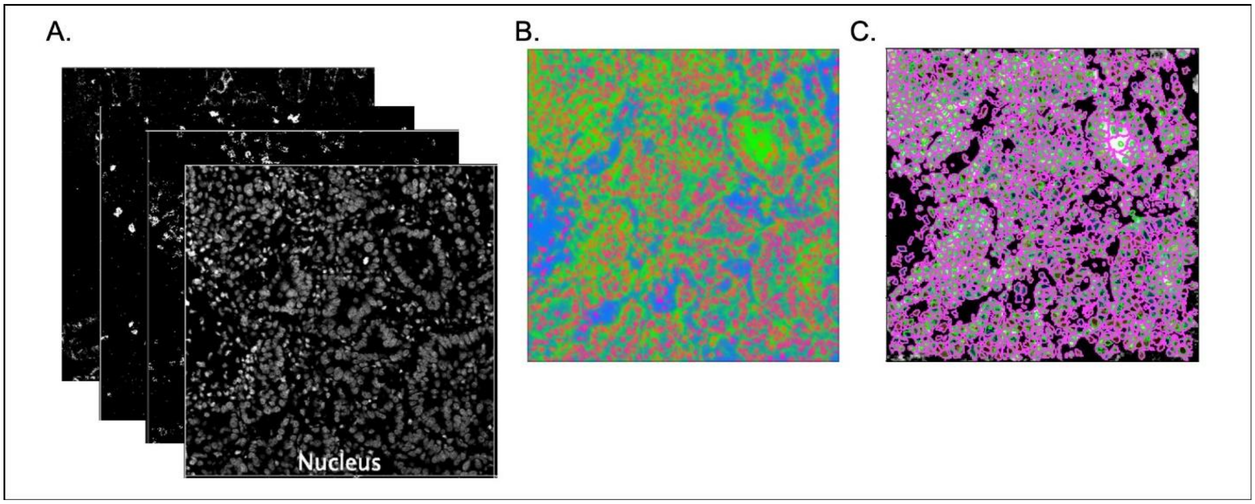

Supplemental Figure 3. Cell segmentation. A) The combination of markers for cell membrane and cell nuclear; **B)** The probability RGB image for cell nuclear (red), cell membrane (green), and background (blue); **C)** the cell segmentation analysis result overlaps with cell membrane probability image. The green line labels the nuclear and the purple line separates the cells.

Supplemental Table 1. Treatment table

Sample name	Immunotherapy	Combination or Monotherapy
S01	Atezolizumab	Monotherapy
S02	Nivolumab	Monotherapy
S03	Atezolizumab	Monotherapy
S04	Pembrolizumab	carboplatin, pemetrexed
S05	Pembrolizumab	Monotherapy
S06	Pembrolizumab	carboplatin, pemetrexed
S07	Nivolumab	Monotherapy
S08	Nivolumab	Monotherapy
S09	Pembrolizumab	Monotherapy
S10	Atezolizumab	Monotherapy
S11	Atezolizumab	Monotherapy
S12	Atezolizumab	Monotherapy
S13	Atezolizumab	Monotherapy
S14	Atezolizumab	Monotherapy
S15	Nivolumab	Monotherapy
S17	Nivolumab	Monotherapy
S18	Nivolumab	Monotherapy
S19	Nivolumab	Monotherapy
S20	Pembrolizumab	Monotherapy
S21	Atezolizumab	Monotherapy
S22	Atezolizumab	Monotherapy
S23	Pembrolizumab	Monotherapy
S24	Pembrolizumab	carboplatin, pemetrexed
S25	Pembrolizumab	Monotherapy
S26	Pembrolizumab	carboplatin, pemetrexed

Supplemental Table 2. Immunotherapy responses

Immunotherapy	PD	PR	p-value
Atezolizumab	5 (41.7%)	4 (30.8%)	0.85

Nivolumab	3 (25.0%)	4 (30.8%)
Pembrolizumab	4 (33.3%)	5 (38.5%)

Supplemental Table 3. Genomic Variation Associated with Response Status

		PD	PR	p-value*
**TP53**	Negative	6 (60.0%)	8 (61.5%)	1
	Mutated	4 (40.0%)	5 (38.5%)	
**LRP1B**	Negative	1 (25.0%)	4 (80.0%)	0.33
	Mutated	3 (75.0%)	1 (20.0%)	
**SPTA1**	Negative	4 (100.0%)	4 (80.0%)	1
	Mutated	0	1 (20.0%)	
**ARID1B**	Negative	3 (75.0%)	5 (100.0%)	0.906
	Mutated	1 (25.0%)	0	
**SMARCA4**	Negative	2 (40.0%)	6 (100.0%)	0.122
	Mutated	3 (60.0%)	0	
**MLL3**	Negative	3 (75.0%)	4 (80.0%)	1
	Mutated	1 (25.0%)	1 (20.0%)	
**STK11**	Negative	8 (80.0%)	13 (100.0%)	0.347
	Mutated	2 (20.0%)	0	
**EPHA3**	Negative	3 (75.0%)	6 (100.0%)	0.83
	Mutated	1 (25.0%)	0	
**KEAP1**	Negative	2 (40.0%)	6 (100.0%)	0.122
	Mutated	3 (60.0%)	0	
**NKX2_1**	Negative	3 (75.0%)	5 (83.3%)	1
	Mutated	1 (25.0%)	1 (16.7%)	
**MLL**	Negative	3 (75.0%)	5 (100.0%)	0.906
	Mutated	1 (25.0%)	0	
**FAT1**	Negative	4 (100.0%)	3 (60.0%)	0.53

	Mutated	0	2 (40.0%)	
**ATM**	Negative	9 (100.0%)	10 (76.9%)	0.358
	Mutated	0	3 (23.1%)	
**NF1**	Negative	4 (100.0%)	5 (83.3%)	1
	Mutated	0	1 (16.7%)	
**CDKN2AB**	Negative	4 (66.7%)	6 (100.0%)	0.439
	Mutated	2 (33.3%)	0	
**TSC2**	Negative	5 (83.3%)	6 (85.7%)	1
	Mutated	1 (16.7%)	1 (14.3%)	
**TET2**	Negative	3 (75.0%)	6 (100.0%)	0.83
	Mutated	1 (25.0%)	0	
**ARID2**	Negative	3 (75.0%)	4 (80.0%)	1
	Mutated	1 (25.0%)	1 (20.0%)	
**CDKN2A**	Negative	8 (88.9%)	12 (92.3%)	1
	Mutated	1 (11.1%)	1 (7.7%)	
**BRCA2**	Negative	9 (100.0%)	12 (92.3%)	1
	Mutated	0	1 (7.7%)	
**MYC**	Negative	7 (87.5%)	8 (100.0%)	1
	Mutated	1 (12.5%)	0	
**ARID1A**	Negative	7 (87.5%)	4 (57.1%)	0.459
	Mutated	1 (12.5%)	3 (42.9%)	
**AR**	Negative	7 (87.5%)	7 (100.0%)	1
	Mutated	1 (12.5%)	0	
**MLL2**	Negative	3 (75.0%)	5 (100.0%)	0.906
	Mutated	1 (25.0%)	0	
**PIK3CA**	Negative	8 (88.9%)	13 (100.0%)	0.85
	Mutated	1 (11.1%)		
**PRKDC**	Negative	4 (80.0%)	5 (100.0%)	1
	Mutated	1 (20.0%)	0	

**NOTCH1**	Negative	9 (100.0%)	12 (92.3%)	1
	Mutated	0	1 (7.7%)	
**EGFR**	Negative	11 (91.7%)	12 (92.3%)	1
	Mutated	1 (8.3%)	1 (7.7%)	
**APC**	Negative	9 (90.0%)	13 (100.0%)	0.893
	Mutated	1 (10.0%)	0	
**FANCA**	Negative	6 (100.0%)	6 (85.7%)	1
	Mutated	0	1 (14.3%)	

*Chi-square test. **PD**: progression of disease. **PR**: partial response.

Supplemental Table 4. KRAS Variation Subtypes with Response Status

KRAS Variations	PD	PR	p-value*
**G12A**	0	2 (15.4%)	0.485
**G12C**	5 (41.7%)	3 (23.1%)	
**G12D**	2 (16.7%)	2 (15.4%)	
**G12V**	2 (16.7%)	2 (15.4%)	
**Q61H**	0	2 (15.4%)	
**Q61L**	1(8.3%)	0	
**others**	2 (16.7%)	2 (15.4%)	

*Chi-square test. **PD**: progression of disease. **PR**: partial response.

Supplemental Table 5. Antibody Panel

Target	Metal	Material ID*
**CD3**	170Er	3170019D
**CD4**	156Gd	3156033D
**CD8**	162Dy	3162035D
**FOXP3**	155Gd	3155016D
**CD68**	159Tb	3159035D
**Arginase-1**	164Dy	3164027D
**CD33**	145Nd	3145017D
**HLA-DR**	174Yb	3174023D
**PanCK**	148Nd	3148020D
**PD-1**	165Ho	3165039D
**PD-L1**	150Nd	3150031D

*Fluidigm Corporation (South San Francisco, CA). **PD**: progression of disease. **PR**: partial response.

Supplemental Table 6. Cell population by type and percentage

Cell Type	Cell Count	Percentage
CD4 T Cell	1409	6.6%
CD68	4397	20.7%
CD68+CD33+PDL1	451	2.1%
CD68+PanCK	623	2.9%
CD68+PDL1	1501	7.1%
CD8 T Cell	3326	15.7%
Foxp3+ Treg Cell	802	3.8%
PanCK	1720	8.1%
PanCK+CD33+Foxp3	4790	22.6%
PanCK+CD68+CD33+PDL1+Foxp3	950	4.5%
PanCK+PDL1	1555	7.3%
PanCK+PDL1+CD33	980	4.6%
weak CD68	4697	22.1%
others	13	0.1%
